# Clinical Insight into Congenital Hypothyroidism Among Children

**DOI:** 10.3390/children12010055

**Published:** 2025-01-03

**Authors:** Hüseyin Anıl Korkmaz

**Affiliations:** 1 Department of Pediatrics, Division of Pediatric Endocrinology, Izmir Faculty of Medicine, University of Health Sciences, 35210 Izmir, Turkey; huseyinanil.korkmaz@sbu.edu.tr; Tel: +90-(0232)-411-6000; 2 Department of Pediatrics, Division of Pediatric Endocrinology, Dr. Behçet Uz Pediatric Diseases and Surgery Training and Research Hospital, 35210 Izmir, Turkey

**Keywords:** congenital, hypothyroidism, newborn, thyroid gland

## Abstract

**Highlights:**

**What are the main findings?**

- Transient congenital hypothyroidism in newborns is characterized by low serum T4 and high serum TSH concentrations, and is caused by iodine deficiency in in iodine-deficient geographic regions.
- Transient hyperthyrotropinemia cases develop secondary to perinatal iodine exposure, TSH and TSH receptor defects, mildly defective intrathyroidal iodine synthesis, hemihypothyroidism, and adjustment disorders in the TSH feedback system.

**What is the implication of the main finding?**

- If infants have congenital hypothyroidism due to iodine overload or maternal blocking antibodies, their requirement for thyroid hormone replacement may decrease or may not persist in these patients, as the effects of these factors disappear within 6 weeks.
- Treatment should be recommended when the FT4 level is in the upper half of the reference range and the TSH level is >20, and treatment should be given when the FT4 level is in the lower half of the reference range and the TSH level is >20 in patients from the newborn screening program.

**Abstract:**

Molecular, genetic, and technological advances have led to increased knowledge regarding neonatal thyroid hormone metabolism disorders. Maternal and fetal hypothyroidism, which can cause psychomotor dysfunction syndromes or low IQ levels, can lead to brain damage, reduced fetal growth and incidental fetal death. The treatment of congenital hypothyroidism detected by screening programs performed during the neonatal period provides normalization of growth, IQ levels, and the physical, mental, and motor development of infants. Therefore, the early diagnosis and treatment of congenital hypothyroidism will prevent the development of complications. In this article, the classification and management of neonatal thyroid diseases are reviewed in light of the current scientific literature.

## 1. Introduction

Clinical and experimental research on the thyroid gland has led to a significant increase in knowledge regarding the ontogenesis, functions and diseases of the neonatal thyroid gland [1]. Congenital hypothyroidism is a disease that can be easily prevented with early diagnosis and treatment. However, if diagnosis and treatment are delayed, this disease can lead to severe delays in mental development, growth, and puberty [2]. Screening programs for congenital hypothyroidism among newborns have been implemented in many countries [1]. Knowledge about fetal and perinatal thyroid function has led to significant progress in the management of thyroid disease in premature and term infants [3]. In this study, the classification and management of neonatal thyroid diseases are reviewed in light of the current scientific literature.

### 1.1. Classification of Congenital Hypothyroidism

Congenital hypothyroidism occurs as a result of the underdevelopment of the thyroid gland, inadequate hormone synthesis, or unresponsiveness to thyroid hormone [4]. This disease occurs equally in boys and girls (male/female: 1:1), but it is detected more frequently in Caucasians [1,2]. The incidence of congenital hypothyroidism varies across countries (Table 1) [5,6,7,8,9,10]. The highest incidence of congenital hypothyroidism was reported in Finland (1/2637), whereas Hungary had the lowest incidence (1/5470) [9,10,11,12]. In Turkey, between 2008 and 2010, when the thyroid-stimulating hormone (TSH) concentration cutoff for congenital hypothyroidism was 7.5 or 10 mIU/L, the incidence of this disease was reported to be 1/650, which is a 4-fold increase compared with the incidence in Finland [9,10,11,12]. The cut-off limit in Finland has varied between TSH 25 and 40 mU/l for the initial screening and between TSH 20 and 40 mU/l for the confirmatory screening [10]. The screening test for primary CH is umbilical cord blood TSH (mU/l) and coverage being near to 100%, practically all the children born in Finland are screened [11,12]. The incidence of congenital hypothyroidism is even greater in patients with Down syndrome [13].

**Figure 1:**
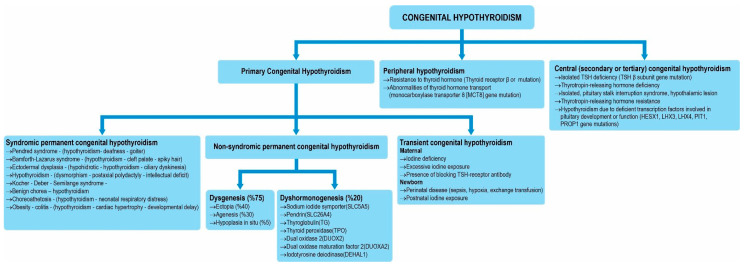
Congenital hypothyroidism classification.

Congenital hypothyroidism is classified into three groups: primary hypothyroidism, peripheral hypothyroidism, and central congenital hypothyroidism (Figure 1) [1,2,14,15,16,17,18]. While the serum free thyroxin (FT4) concentration is relatively low in individuals with central congenital hypothyroidism, serum TSH levels may be low, normal, or slightly elevated [1,2]. Other pituitary hormones should be investigated in patients with congenital hypothyroidism, and thyroxine therapy should not be started without ruling out adrenal insufficiency. Primary congenital hypothyroidism can be further classified into three groups: syndromic permanent hypothyroidism, nonsyndromic permanent hypothyroidism, and transient congenital hypothyroidism (Figure 1) [1,2,14,15,16,17,18].

Nonsyndromic permanent congenital hypothyroidism can be classified into two subgroups: thyroid dysgenesis and dyshormonogenesis [1,2,14,15,16,17,18]. Ectopic thyroid, thyroid hypogenesis, and thyroid agenesis are three types of thyroid dysgenesis. Ectopic thyroid dysgenesis accounts for 60% of all thyroid dysgenesis cases [14,15,16,17,18]. The thyroid gland develops in the tongue cavity during embryonic life, and abnormal problems that occur during its migration to its normal anatomical location cause ectopic thyroid disease [19]. Sometimes, the thyroid gland may migrate further and settle in the substernal region; alternatively, it may remain in the olfactory cavity of the tongue without migrating to its final destination. Ectopic thyroid tissue can be detected via thyroid scintigraphy. In the past, congenital hypothyroidism patients were divided into two groups: those with and without severe hypothyroidism. Hypothyroidism is believed to cause dyshormogenesis, whereas nongoitrous hypothyroidism causes dysgenesis. Nongrogenic hypothyroidism is believed to be sporadic, however, it is now recognized that thyroid dysgenesis is not sporadic and that many genes are involved in its etiology. The pathogenesis of thyroid dysgenesis can be explained by Mendelian or non-Mendelian inheritance [11,12]. While the disease usually occurs sporadically, familial cases of thyroid dysgenesis are associated with homeobox genes, including TTF-1, TTF-2, PAX-8 and TSHR [15,16,17,18]. GL153 transcription factor mutations may be associated with neonatal diabetes, congenital hypothyroidism, and congenital glaucoma [20].

### 1.2. Thyroid Dyshormonogenesis

Thyroid hormones are predominantly synthetized from iodine and tyrosine. Iodine enters thyroid follicle cells via facilitated diffusion through the sodium/iodide symporter, which is regulated mainly by TSH [21,22]. From follicle cells, TSH enters the colloid space via the pendrin protein. Mutations in the pendrin protein induce a type of genetic hearing loss called Pendred syndrome, however, mutations that do not result in hearing loss have also been described [21,22]. After binding to TSH receptors in thyroid follicle cells, TSH increases the serum cAMP level and accelerates synthesis of thyroglobulin. Thyroglobulin synthesized in the endoplasmic reticulum transports tyrosine into the colloid space as a carrier protein. In the colloid space, iodine loses its electrons and is oxidized by the action of the thyroid peroxidase enzyme [21,22]. Subsequently, oxidized iodine combines with tyrosine via the action of the thyroid peroxidase enzyme to form monoiodotyrosine and diiodotyrosine. Monoiodothyrosines and diiodothyrosine combine to synthesize T3 and T4. Reverse T3 (rT3) is formed from monoiodothyrosine and diiodothyrosine as an adaptation mechanism. Thyroglobulin acts as a shuttle by transporting T3 and T4 from the colloid space into follicle cells. T3 and T4 released by enzyme proteases pass into the capillary circulation [21,22].

One-third of congenital hypothyroidism occurs as a result of dyshormonogenesis, which is a functional disease of the orthotopic thyroid gland [14,15,16,17,18]. In patients with dyshormonogenic disorders of the thyroid gland, consanguineous marriages are common, and multiple members of the family are affected [14,15,16,17,18]. Since consanguineous marriages are more common in Turkey and Middle Eastern countries, thyroid dyshormonogenesis accounts for half of all cases of congenital hypothyroidism. Decreased iodine intake, organification defects related to iodine intake, thyroglobulin abnormalities, iodotyrosine deiodinase, and recycling deficiency are observed in patients with dyshormonogenic goiter [14,15,16,17,18]. The clinical findings of dyshormonogenetic goiter are similar to those of congenital hypothyroidism, except for the development of goiter in affected patients [14,15,16,17,18]. In a study conducted in the United Arab Emirates, 65 patients with congenital hypothyroidism were evaluated, and dyshormogenesis was found in two-thirds of the patients because of frequent consanguineous marriages [16]. Since dyshormogenesis is observed more frequently in societies with consanguineous marriages, an increased incidence of dyshormogenesis was found in their study population [16].

#### 1.2.1. TSH Resistance

The TSH receptor is a member of the G protein-coupled receptor family. The TSH receptor gene is located on chromosome 14, and the extracellular hormone binding region is encoded by 9 exons [23,24]. Exon 10 encodes seven transmembrane and intracellular regions of the receptor. Germline mutations of the thyrotropin receptor gene have recently been associated with congenital or acquired thyroid disease [23,24]. Defects in this gene cause asymptomatic hyperthyrotropinemia. In some cases of congenital hypothyroidism, a lack of iodine uptake and thyroid hypoplasia have been reported. TSH resistance is characterized by a decreased response to TSH in the thyroid gland, increased serum TSH levels, a normal FT4 ratio and hypoplasia or a normal thyroid gland [23,24]. Patients may present with subclinical hypothyroidism or congenital hypothyroidism [23,24]. In Arab countries, TSH resistance mutations are frequently observed due to the high incidence of parental consanguinity [18]. In newborns with normal serum FT4 and elevated TSH levels, TSH resistance should be considered first in cases of consanguineous marriage and when elevated TSH levels are observed in siblings and close relatives. Most patients with heterozygous TSH resistance do not experience delays in mental development, motor function, growth, or puberty. Large case series have revealed that patients with heterozygous TSH receptor variants do not require thyroid hormone replacement, but patients with homozygous variants may require thyroid hormone replacement. Although serum TSH levels are increased in these patients, thyroid volume does not increase in proportion to TSH. DUOX2 mutation should be considered after TSH resistance is ruled out by genetic analysis. DUOX2 is found in the apical membrane of the thyroid gland and causes H2O2 formation, which is required for the activity of the thyroid peroxidase enzyme [25]. In a clinical study evaluating 45 patients with congenital hypothyroidism, the prevalence of DUOX2 pathogenic variants was 29% [25]. Some patients with DUOX2 mutations may present with goiter.

#### 1.2.2. Defects in the Sodium/Iodide Symporter

The transportation of iodide from the plasma to the cytosol across the thyroid follicle cell membrane is the first step in thyroid hormone synthesis [26,27]. Patients may present with congenital hypothyroidism, and lifelong thyroid hormone replacement therapy is needed [26]. In the presence of a goiter, the diagnosis is made by the absence of radioactive iodine uptake and increased serum TSH levels. Circulating iodine is not concentrated in other iodine-concentrating tissues, such as the salivary glands and gastric mucosa. Lugol’s solution improves hypothyroidism by increasing serum iodine levels [26,27]. In the literature, a homozygous missense and loss-of-function T354P [Thr354-->Pro (ACA-->CCA)] mutation in the sodium/iodide symporter gene was detected in 2 Japanese patients [27].

### 1.3. Peroxidase System Deficiency

Iodine taken up by the thyroid gland is oxidized, and thyroglobulin-bound tyrosyl residues are iodized to form monoiodotyrosine and diiodotyrosine [14,15,16,17,18,23]. One monoiodotyrosine and diiodotyrosine form T3, and two diiodotyrosines form T4. Iodization and tyrosyl coupling are catalyzed by the thyroid peroxidase enzyme system associated with thyroid NADPH oxidase [14,15,16,17,18,28]. Defects in the thyroid peroxidase enzyme system include qualitative deficiency of thyroid peroxidase, abnormal and defective thyroid peroxidase, or deficiency of hydrogen peroxide formation [11,12,13,23]. A complete defect in the thyroid peroxidase enzyme system can be detected by the perchlorate discharge test. In this test, radioactive iodine and perchlorate are administered. If thyroid iodide (TI) is defective, radioactive iodine taken up by the thyroid is rapidly discharged. A study in China examined 192 patients with congenital hypothyroidism; the prevalence of thyroid peroxidase (TPO) enzyme system gene defects was 1% [28]. In another study conducted in China, a novel TPO variant (c.1682C > T/p. T561M) was discovered [29]. In patients with thyroid peroxidase gene mutations, lower FT3, lower FT4, increased TSH and markedly increased serum thyroglobulin levels were detected depending on the magnitude of change in the single gene variant. On thyroid ultrasonography, patients may present with goiter associated with thyroid volumes above 2 SDSs according to their age.

#### Pendred Syndrome

Pendrin is a multifunctional anion exchanger expressed in the thyroid, inner ear, and kidney; in the thyroid, pendrin is located in the apical membrane of follicular cells and is thought to mediate apical iodide transport [17]. Pendred syndrome is caused by homozygous or compound heterozygous pathogenic variants in the SLC26A4 gene, which encodes the pendrin gene. This disease occurs as a result of a defect in the Pendrin gene, which is called the chlorine–iodine transport protein [30]. Pendred syndrome is inherited as an autosomal recessive trait. It may cause familial goiter and deafness due to damage to the eighth cranial nerve [28]. In a study conducted in China, 192 patients with congenital hypothyroidism were evaluated, and the prevalence of pathologic variants of the SLC26A4 gene was 4% [29].

### 1.4. Defective Thyroglobulin Synthesis

Thyroglobulin is a glycoprotein synthesized within the endoplasmic reticulum (ER) of thyroid follicular cells; it forms exocytotic vesicles that migrate into the colloid via the apical membrane [21]. The tyrosine residues on thyroglobulin are used as substrates for the synthesis of thyroid hormone [22]. Genetic defects lead to deficiency and structural abnormalities in thyroglobulin synthesis, with an incidence rate of 1/80,000 [14,15,16,17,18,30]. Goiter and hypothyroidism are present at birth among individuals with thyroglobulin synthesis defects [22]. The onset of mild defects occurs at a later date [27]. Defects in the thyroid peroxidase enzyme and thyroglobulin genes constitute the two most common causes of dyshormonogenesis [21,22,30]. In patients with thyroglobulin gene mutations, the serum thyroglobulin level decreases to almost zero, the levels of FT3 and FT4 decrease, and the TSH level increases depending on the degree of the change in a single gene variant.

### 1.5. Iodotyrosine Deiodinase Deficiency

Iodotyrosine deiodinase (DEHAL1 or IYD) is an important enzyme in iodine balance and plays a role in iodine recycling [31]. It liberates iodine from its known substrates monoiodotyrosine or diiodotyrosine. The importance of this step is demonstrated by the consequences of a genetic DEHAL1 malfunction. Children with DEHAL1 pathogenic variants have an elevated loss of iodine through the urine in the form of MIT and DIT and may suffer from hypothyroidism, compressive goiter and mental retardation [21]. It is inherited as an autosomal recessive trait. Its deficiency results in early onset as well as rapid uptake and spontaneous clearance of radioactive iodine [31]. Most thyroidal radioactive iodine is discharged within 48 h. Increased serum iodotyrosine levels are detected in patients with iodotyrosine deiodinase deficiency. Positive effects of iodide supplementation on thyroidal iodide uptake, serum T4 levels and goiter size have been observed in young patients [31]. Besides in genetic iodotyrosine deiodinase deficiency, elevated levels of DIT in serum are also found in inflammation and infection [31]. The affected individuals in the few families identified and molecularly characterized so far were homozygous for missense or frame shift mutations, dramatically reducing DEHAL1 enzymatic activity.

## 2. Peripheral Hypothyroidism

### 2.1. Thyroid Hormone Resistance Syndrome

Thyroid hormone resistance syndrome is a rare disorder that usually shows autosomal dominant inheritance [32,33]. Patients with thyroid hormone resistance usually become euthyroid but may rarely present with signs and symptoms of thyrotoxicosis or hypothyroidism [32,33]. In patients with thyroid hormone resistance, high FT4 and FT3 levels are accompanied by normal or increased TSH levels [22,31]. Thyroid hormone resistance can be divided into three categories: diffuse resistance, peripheral resistance, and partial pituitary resistance. Partial pituitary resistance to thyroid hormones is a very rare cause of hyperthyroidism. Partial hypophyseal resistance is characterized by thyroid hormone resistance in the pituitary gland without any evidence of resistance in peripheral tissues. Thyroid hormone resistance manifests clinically as hyperthyroidism or hypothyroidism [32,33]. Thyroid hormone resistance syndrome may be overlooked because there is insufficient information on this entity, which has nonspecific clinical signs and symptoms. Mutations in the thyroid hormone receptor gene, which encodes the TRβ gene, are found in 85% of patients with thyroid hormone resistance. Thyroid hormone receptor proteins are encoded by two genes. Each gene has two transcripts, TRα1, TRα2, TRβ1, and TRβ2 [32,33]. TRα receptor mutations may present with growth retardation, mild or moderate mental retardation, skeletal dysplasia, and constipation [33]. The thyroid hormone profile of this receptor consists of anemia, increased creatine kinase enzyme levels, hypercholesterolaemia, normal TSH levels, low FT4 levels, and high FT3 levels [33].

### 2.2. Thyroid Hormone Transporter Deficiencies

The transport of thyroid hormones is mediated by sodium-dependent transporter polypeptides, sodium-independent organic anion transporters, L-type amino acid transporters and monocarboxylate transporter 8 [34]. Monocarboxylate 8 gene mutations cause thyroid and neurologic dysfunction [34]. These patients present with hypotonia, poor head control, athetoid and dystonic movements, hyperreflexia, nystagmus and severe delays in mental development in infancy and childhood. Serum T3 levels are high, serum T4 and FT4 levels are low, and serum TSH levels are within their normal ranges or increased among patients with thyroid hormone transporter deficiencies [34]. Allan–Herndon–Dudley syndrome is an X-inherited mental retardation syndrome that occurs as a result of a mutation in the MCT-8 gene.

## 3. Transient Congenital Hypothyroidism

**Figure 2:**
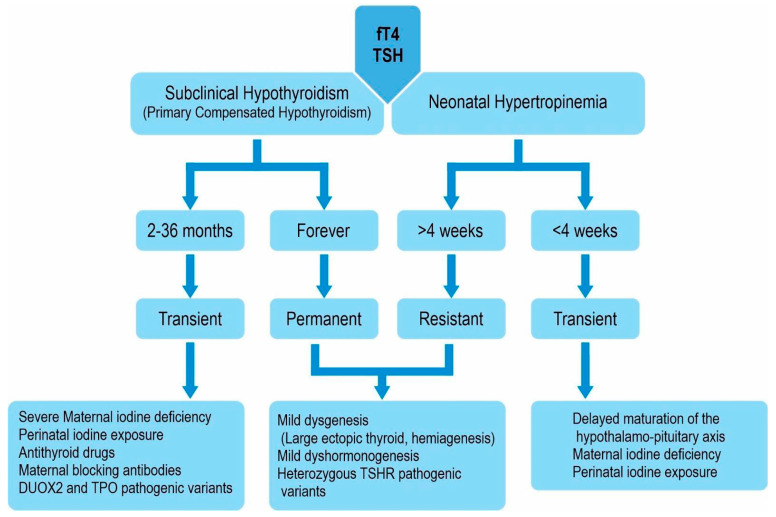
Approach to neonatal hypertropinemia.

Transient congenital hypothyroidism (TCH) in newborns is characterized by low serum T4 and high serum TSH concentrations and is caused by iodine deficiency. Transient hypothyroidism is observed in 15–20% of premature infants in some parts of Europe where iodine deficiency is observed [1,5,35]. Premature infants need more iodine than term infants do to ensure normal T4 levels, therefore, transient iodine deficiency may develop in iodine-deficient geographic regions [1,5,35]. Transient hypothyroidism develops in the first two weeks of extrauterine life and masks the transient hypothyroxinemia associated with prematurity (Figure 2). Urinary and thyroid gland iodine concentrations are decreased [1,5,10,30]. Premature infants are susceptible to the inhibitory effects of iodine overload due to an iodine overdose [1,5,10,35].

The fetal thyroid is sensitive to the inhibitory effects of iodine on thyroid hormone synthesis. Excessive maternal dietary iodine intake, the use of iodine-containing medications, or the use of iodine-containing disinfectants for skin cleaning cause excessive iodine exposure in newborns (Figure 2) [1,5,35]. Additionally, some drugs (dopamine and amiodarone) affect iodine metabolism.

Transient congenital hypothyroidism (TCH) occurs in 5–10% of infants who participate in newborn screening programs [1,5,10,35]. In North America, the most common causes of TCH are goitrogenic agents and maternal TSH receptor-blocking antibodies [1,5,35]. Antibody-mediated congenital hypothyroidism occurs at an incidence rate of 1–2%. Transient congenital hypothyroidism is more common in regions with endemic iodine deficiency and is caused by iodine deficiency associated with increased thyroid hormone requirements in the neonatal period [1,5,35].

Maternal iodine or antithyroid drug intake should be considered in all patients with congenital hypothyroidism [1,5,35]. The presence of goiter in infants is a supportive finding of transient hypothyroidism induced by drugs or goitrogens [1,5,35].

Additionally, congenital hypothyroidism due to maternal iodine deficiency may be observed in these patients [36]. The daily iodine requirements are 8 μg/kg in newborns and 30 μg/kg in preterm newborns [36]. Since 100 mL of breast milk contains 5 μg of iodine, in the case of maternal iodine deficiency, these newborns are prone to suffer from iodine deficiency [36]. If the newborns of these mothers receive limited amounts of thyroid hormone through feeding, they will have moderately high serum FT3, increased TSH, thyroglobulin, and decreased FT4 levels. In these patients, replacement therapy with Na-L-T4 and iodine should be administered [1,5,35,36]. A spot urine iodine test is recommended for infants with elevated TSH from neonatal thyroid hormone screening to rule out iodine deficiency or excess. In infants with iodine deficiency, the serum thyroglobulin level is high, while the serum FT3 level is high and the FT4 level is low, therefore, spot urine iodine excretion is helpful for diagnosis. The exposure of the episiotomy incisions to iodine after the use of batikon for 10 days causes transient neonatal hypothyroidism. Therefore, it is recommended that spot urine iodine tests be performed at all centers.

The thyroid hormone levels of patients with resistant TSH elevation or transient CH may increase during adolescence and pregnancy, as observed in newborns and infants, therefore, these patients may develop hypothyroidism and goiter [1,5,35,36]. Since these patients may require retreatment, long-term follow-up is recommended. Owing to the launch of the CH screening program worldwide and the use of low TSH threshold values for screening tests, pediatric endocrinologists have encountered a large group of patients with mild thyroid dysfunction. New approaches are needed for the evaluation and management of patients with transient and permanent CH.

## 4. Euthyroid Hyperthyrotropinemia

Asymptomatic hyperthyrotropinemia is a very common disease and may have transient or permanent manifestations (Figure 2). Transient hyperthyrotropinemia occurs at a rate of 1:8000 in Europe, and 50% of cases develop secondary to perinatal iodine exposure [1,2,3,4,5,30]. Other causes include TSH and TSH receptor defects, mildly defective intrathyroidal iodine synthesis, hemihypothyroidism, and adjustment disorders in the TSH feedback system [1,2,3,4,5,10,35]. Germline mutations in the thyrotropin receptor gene are associated with asymptotic hyperthyrotropinemia. Most cases are caused by combined gene mutations [35]. Elevated TSH persists in 1/3 of patients, with elevated TSH detected during newborn screening [1,2,3,4,5,10,35]. Thyroid dysgenesis and TSH receptor resistance play a role in the etiology of these cases. These cases should not be called subclinical hypothyroidism; instead, they should be termed isolated TSH elevation.

In patients with isolated TSH elevation, inaccurate results indicating increased TSH levels may be due to laboratory interference [37]. The avoidance of laboratory interference is the responsibility of clinical biochemists, so clinical biochemists should alert clinicians regarding this issue. Although clinical biochemists in developed countries can warn clinicians about laboratory interference, clinicians in developing countries should be careful about laboratory interference [37]. In patients with asymptomatic hyperthyrotropinemia without any clinical symptoms of hypothyroidism, TSH cannot be suppressed by thyroid hormone treatment, so macro-TSH should be considered [37]. In these macro-TSH patients, falsely elevated TSH levels with normal thyroid ultrasonograms are observed. Patients receiving biotin treatment due to biotinidase deficiency may exhibit high FT4 and low TSH levels in clinical practice [37]. In addition, heterophile antibodies may cause laboratory interference in thyroid function tests. If the results of serum thyroid function tests and clinical findings are not consistent, the possibility of interference/artifacts should always be considered.

## 5. Clinical Findings and Diagnosis

Patients with positive screening results are evaluated according to their medical history, physical examination results, and laboratory findings. During the diagnostic process, thyroid function tests (FT3, FT4, TSH, thyroglobulin, and thyroid antibodies), scintigraphy and then USG should be performed under optimal conditions before or within 1 week after starting treatment [1,5,35]. Serum thyroglobulin levels provide information about the etiology of congenital hypothyroidism. Serum thyroglobulin levels close to zero suggest thyroid agenesis, low levels suggest thyroid hypoplasia, and high levels suggest dyshormonogenesis.

The clinical manifestations of congenital hypothyroidism (CH) are not obvious at birth, and most newborns are undiagnosed [1,2,3,4,5]. The main reason for this uncertainty is the protective effect of maternal T4 passing through the placenta to the infant. Approximately 50% of the T4 in cord blood is maternal in origin [1,2,3,4,5]. In addition, most infants with CH, except those with thyroid agenesis, produce low levels of T4. Thus, overt clinical symptoms develop slowly, which emphasizes the importance of diagnosis by screening tests and the initiation of treatment within the first two weeks. On the other hand, it is possible for infants with CH not to undergo screening tests. Therefore, overlooked clinical signs can lead to irreversible neurological damage. Omer Tarim et al. published the largest series of congenital hypothyroidism cases with delayed diagnosis in the literature before the implementation of newborn thyroid screening tests in Turkey [38]. The mean age at diagnosis was 49.22 months. All 1000 patients who were diagnosed with congenital hypothyroidism between 1972 and 1992 had delayed mental development. More than half (55.4%) of these patients were diagnosed after the age of two, and 3.1% of them were diagnosed during the neonatal period. The presenting complaints were delayed growth in 26.7% of the patients, inability to speak in 21.4% of the patients and inability to walk in 18.1% of the patients [38]. According to the clinical symptoms and findings, hypotonia was detected in 72% of the patients, constipation was detected in 66.8% of the patients, and cretinoid faces and macroglossia were detected in 64.6% of the patients. Some patients with delayed diagnosis exhibit hypotonia and die before diagnosis [38]. In addition, increased lumbar lordosis is a sign of hypotonia. Although 64.6% of these patients had a cretinoid face, some patients may not have this characteristic feature. These patients can be diagnosed through congenital screening programs. Babies with CH have a normal weight and height at birth, but their head circumference may be slightly greater [1,2,3,4,5]. They have large anterior and posterior fontanelles, and observation of this parameter at birth may be the first sign for early diagnosis of congenital hypothyroidism. Only 3% of normal newborn babies have a posterior fontanelle larger than 0.5 cm. Therefore, serum thyroid stimulating hormone (TSH) and free T4 (FT4) levels should be measured in babies born with a large posterior fontanelle [1,2,3,4,5]. To eliminate the possibility of central hypothyroidism, thyroid function tests should be performed for every baby whose neonatal jaundice is prolonged for more than 3 weeks. In addition, TSH may increase at a later stage in low birth weight babies. Serum FT4 and TSH measurements should be performed in babies with a history of excessive sleep (lethargy), feeding difficulties and constipation and in babies with an umbilical hernia, cold and mottled skin appearance, large fontanelle, rough face, large tongue, coarse-muffled crying, hypotonia, or a goiter detected on physical examination. However, a detailed examination of patients with cogenital hypothyroidism should be performed, especially to detect concomitant cardiac anomalies and dysmorphic findings [1,2,3,4,5]. The presence of thyroid disease, goiter or a known genetic disease in the family, especially in mothers and in cases of consanguineous marriages, should be considered. Transient neonatal hyperthyrotropinemia in the sibling and a history of autoimmune thyroiditis in the mother may indicate the presence of blocking antibodies.

Knee radiography is not a method used in the diagnosis of congenital hypothyroidism. The absence of femoral and tibial epiphyses detected on direct radiographs indicates the severity of intrauterine hypothyroidism [1,2,3,4,5]. On the basis of the ossifications detected in normal knee radiographs, the distal femoral epiphysis formed at 36 weeks, and the proximal epiphysis of the tibia formed at 38 weeks of gestation. In normal term newborns, two ossification centers should be located in the knee. Hand radiography was performed starting at 3 months of age.

**Figure 3:**
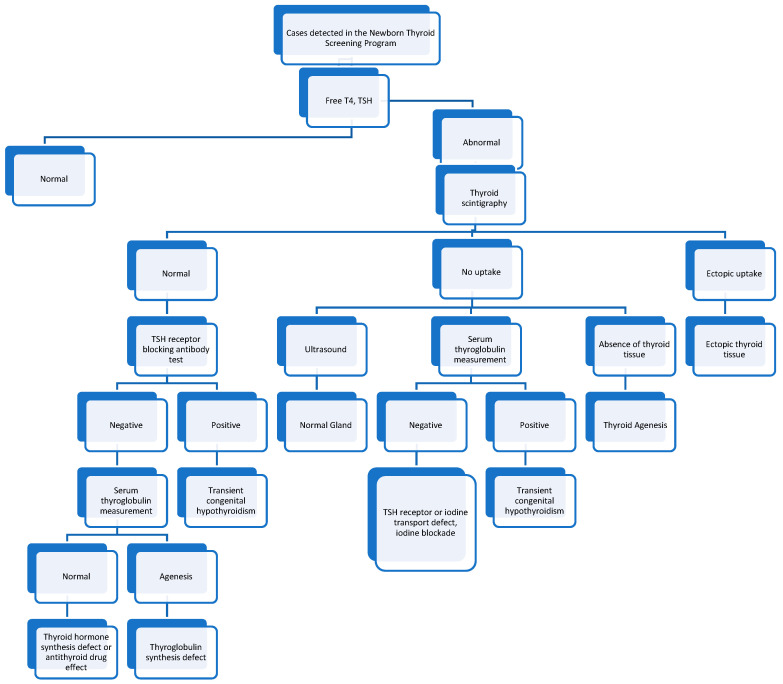
Algorithm for cases detected in the newborn thyroid screening program. T4: free thyroxine, TSH: thyroid stimulating hormone.

The diagnosis of congenital hypothyroidism is based on the detection of increased serum TSH and decreased serum T4 and FT4 levels [39]. If the results of serum thyroid function tests do not match the clinical findings of patients, the possibility of interference/artifacts should always be considered [37,39]. In a study evaluating term infants with congenital hypothyroidism, thyroid scintigraphy performed before the start of treatment allowed for the early diagnosis of patients with ectopic hypothyroidism and agenesis and provided more information about permanent hypothyroidism [40]. The serum TSH level at diagnosis and the mean levothyroxine dose during treatment provide information about permanent hypothyroidism [33]. Serum FT4 and TSH levels should be obtained from patients with abnormalities detected in newborn screening tests (Figure 3). Thyroid scintigraphy should be performed in patients with laboratory and clinical findings of hypothyroidism (Figure 3) [36]. In newborns, iodine-123 (I-123) or sodium pertechnetate 99 m (Tc99m) thyroid scintigraphies are preferred. The iodine 131 (I-131) uptake test is generally not preferred because it exposes the thyroid gland and body of the infant to high doses of radioactivity. Thyroid scintigraphy reveals ectopia or agenesis in the thyroid gland.

When thyroid agenesis is detected via thyroid scintigraphy, the presence of TSH receptor inactivating mutations, thyroid stimulating hormone subunit beta (TSHB) gene mutations, iodine retention defects and maternal thyrotropin receptor blocking antibodies should be considered. The increased retention of radioactive iodine activity in the thyroid tissue and increase in thyroid size detected via thyroid scintigraphy may be one of the causes of dyshormonogenesis other than iodine retention defects. In accordance with the results of the thyroid scintigraphy, ultrasound, and measurement of serum thyroglobulin levels should be performed (Figure 3). The combined use of thyroid scintigraphy and thyroid ultrasound helps elucidate the etiology of congenital hypothyroidism. Transient congenital hypothyroidism, TSH receptor defects, or iodine blockade are diagnosed on the basis of the results of ultrasonographic tests and measurements of serum thyroglobulin levels (Figure 3) [21].

## 6. Treatment

There is a race against time in the treatment of congenital hypothyroidism; every minute of delay causes a decrease in the newborn’s IQ score [38,41]. In cases of congenital hypothyroidism, 2 IQ points are lost every week of delayed treatment [1,38,41]. Therefore, when congenital hypothyroidism is diagnosed, treatment should begin immediately. The thyroid hormone requirements of the central nervous system are met by local T4-T3 conversion. Most (70%) T3 in the cerebral cortex is produced via monoiodinization of local T4 [1,2,3,4,5,39]. Serum T4 and free T4 values should be maintained in the upper half of the normal range to ensure the adequacy of thyroid hormone treatment in infants [1,2,3,4,5,32]. When congenital hypothyroidism is diagnosed, Na-L-T4 should be given orally at a daily dose of 10–15 μg/kg [1,2,3,4,5,39]. From the beginning of therapy of congenital hypothyroidism with levothyroxine (LT4), initially recommended dose of this medication was the 5–7 µg/kg/day; subsequently, the recommend dose was changed to 8–10 µg/kg/day and, most recently, 10–15 µg/kg/day was recommended by the American Academy of Pediatrics [1,39,42,43]. This drug should be taken before feeding with water only. It should not be given simultaneously with other medications. Levothyroxine therapy should not be given with iron medications, vitamin D, antileptic drugs and oral alendronate as this drug may interact with these drugs [1,2,3,4,5,39]. The dose of thyroid hormone given for the treatment of thyroid agenesis is greater than the dose required for thyroid ectopy and hypoplasia. Daily doses of 5–8 μg/kg Na-L-T4 are recommended for mild hypothyroidism, 8–10 μg/kg for moderate hypothyroidism, and 10–15 μg/kg for severe hypothyroidism during newborn period [1,2,3,4,5,39,42,43]. If intravenous levothyroxine treatment is needed, the treatment dose should not exceed 80% of the oral dose [32]. The liquid form of levothyroxine is produced, but it is not available in our country. Levothyroxine tablets can be given in small spoons mixed with breast milk or water. In the first weeks of treatment, serum FT4 and TSH are the most important markers for the management of treatment [1,5,32]. Thyroid function tests should be performed on an empty stomach before the morning dose or 4 h after the last dose, whereas fasting is not necessary for the neonates with congenital hypothyroidism. After thyroid hormone therapy is started, treatment should be monitored 1–2 weeks after the first dose, every 2 weeks until TSH levels return to normal, and every 1–3 months until the age of 1 year.

Table 2 shows the therapeutic decision algorithm for patients from the neonatal screening program. According to our experience with clinical data, follow-up without treatment should be recommended when the FT4 level is in the upper half of the reference range and the TSH level is >20, and treatment should be given when the FT4 level is in the lower half of the reference range and the TSH level is >20 in patients from the newborn screening program.

When evaluating patients referred from neonatal TSH screening programs, the relationship between serum FT4 and TSH levels should be examined, and the decision for follow-up or treatment should be made on the basis of the relationship between serum FT4 and TSH levels. The serum FT4 and TSH levels during follow-up are very important when these patients are being evaluated. While serum FT4 decreases during follow-up, an increase in TSH is very important for therapy-related decisions.

Although we initiated thyroid hormone replacement in patients with congenital hypothyroidism according to their weight, if the thyroid hormone replacement dose decreases even if the infant’s growth and neurodevelopment are very good, these patients are most likely to have transient congenital hypothyroidism. If infants have congenital hypothyroidism due to iodine overload or maternal blocking antibodies, their requirement for thyroid hormone replacement may decrease or may not persist in these patients, as the effects of these factors disappear within 6 weeks.

The growth and development of infants with congenital hypothyroidism return to normal with early and adequate treatment, and the delay in bone maturation detected at the time of diagnosis can be compensated for at 1–2 years of age [31,32]. In most cases of congenital hypothyroidism, with treatment, the IQ and mental and motor development of these infants return to their age-adjusted normal ranges [31,32].

## 7. Conclusions

In families with a history of hereditary disorders of hypothalamic and pituitary function, thyroid hormone metabolism, a history of maternal drug therapy affecting fetal thyroid function, or known maternal autoimmune thyroid diseases, newborns should be evaluated for the potential presence of fetal and neonatal thyroid diseases. Maternal and fetal hypothyroidism, which can cause psychomotor dysfunction syndromes or low IQ levels, are associated with brain damage, reduced fetal growth, and incidental fetal death. Low IQ levels and motor disorders have been reported in severe cases of congenital hypothyroidism [38,39,41]. Therefore, early diagnosis and treatment of maternal and fetal hypothyroidism can significantly reduce the morbidity and mortality rates of these patients.

## Figures and Tables

**Table 1:** Incidence of congenital hypothyroidism by country.

Country	Incidence
Finland	1:2637
Turkey	1:2800
Sweden	1:3298
Scotland	1:3300
France	1:3442
Israel	1:3606
Ireland	1:4012
Greece	1:4200
Australia	2:4253
USA and Canada	1:4464
Denmark	1:5231
Hungary	1:5470

**Table 2:** Therapy decision algorithm.

Levothyroxine Therapy	Free T4	TSH (IU/mL)
Start levothyroxine	Normal	>20
Start levothyroxine	Low	6–20
Start levothyroxine	Lower half of range	6–20 IU/mL—No trend for decreasing
Observation without levothyroxine therapy	Upper half of range	6–20 IU/mL—Trend for decreasing

Free T4: free thyroxine; TSH: thyroid stimulating hormone.

## Data Availability

This publication does not contain any data or images from any individual person.
